# Enhancing Ionic Transport in Green Gel Polymer Electrolytes Based on Alginate/Lignin/Na_2_SO_4_ Systems

**DOI:** 10.1021/acsomega.6c03857

**Published:** 2026-05-21

**Authors:** Renata Moreno Bueno, Lincoln Audrew Cordeiro, Eduarda Vieira Silva, Chiara das Dores do Nascimento, Everton Granemann Souza, Juliano Marini, Neftali Lenin Villarreal Carreno, André Luiz Missio, Rafael de Avila Delucis, Camila Monteiro Cholant, Amanda Dantas de Oliveira

**Affiliations:** † Graduate Program in Materials Science and Engineering (PPGCEM), 37902 Federal University of Pelotas (UFPel), Pelotas 96010-610, Rio Grande do Sul, Brazil; ‡ Graduate Program in Sciences and Technologies in Education (PPGCITED), Federal Institute of Education, Science and Technology of Rio Grande do Sul (IFSul), Pelotas 96060-290, Rio Grande do Sul, Brazil; § Graduate Program in Electronics and Computer Engineering (MEEC), 67714 Catholic University of Pelotas (UCPel), Pelotas 96015-560, Rio Grande do Sul, Brazil; ∥ Graduate Program in Materials Science and Engineering (PPGCEM), Federal University of São Carlos (UFSCar), São Carlos 13565-905, São Paulo, Brazil

## Abstract

Green gel polymer electrolytes (GPEs) based on renewable polymers are promising candidates for sustainable energy storage; however, achieving high ionic conductivity remains a major challenge due to limited salt dissociation and restricted ion mobility. In this work, a biobased GPE system combining sodium alginate, lignin, and sodium sulfate (Na_2_SO_4_) is proposed to enhance ionic transport through the interplay between salt concentration, polymer–ion interactions, and matrix organization. A systematic compositional study was performed to evaluate the effects of Na_2_SO_4_ and lignin incorporation on the electrochemical properties of the system. The ionic conductivity increased from 2.09 × 10^–5^ to 2.91 × 10^–4^ S cm^–1^, with the optimized composition (Alg-Lig-Na20.6) achieving among the highest values reported for alginate-based electrolytes. This enhancement is attributed to an optimal balance between charge carrier density and mobility, while excessive salt content led to ion association and reduced transport efficiency. Dielectric and electric modulus analyses revealed reduced relaxation times and enhanced charge carrier dynamics, while transference number measurements confirmed predominantly ionic conduction (*t*_ion_ up to 0.98). The electrolytes exhibited good electrochemical stability, with a stability window up to 2.85 V and stable cyclic voltammetry response over multiple cycles. These findings demonstrate that lignin incorporation, combined with controlled salt content, is an effective strategy to tailor ionic transport and electrochemical stability in biobased GPEs.

## Graphical abstract

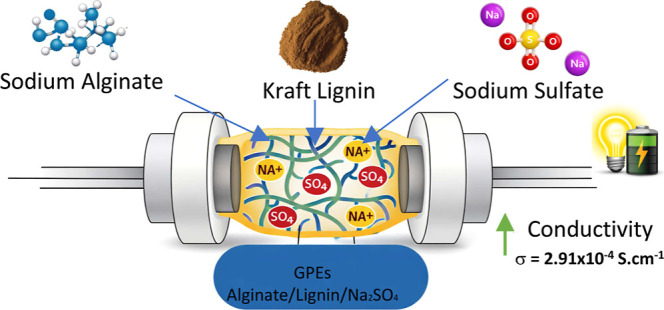

## Introduction

The development of polymer electrolytes for energy storage applications has traditionally relied on synthetic polymer matrices, including widely used systems based on poly­(ethylene oxide) (PEO),^1^ poly­(vinylidene fluoride) (PVDF),^2^ poly­(vinylidene fluoride-*co*-hexafluoropropylene) (PVDF-HFP),^3^ polyacrylonitrile (PAN),^4^ and poly­(methyl methacrylate) (PMMA).^5^ These materials are widely employed due to their good mechanical integrity, dimensional stability, and ease of processing, which enable reliable fabrication and operation in batteries and supercapacitors.^6,7^

However, increasing environmental concerns associated with fossil-based polymer electrolytes have stimulated the development of gel polymer electrolytes (GPEs) based on renewable biopolymers.^8^ Compared with conventional solid polymer electrolytes (SPEs), GPEs generally exhibit higher ionic conductivity due to the presence of a more flexible and partially solvated polymer network, making them promising candidates for sustainable energy storage applications.^9–11^ Nevertheless, biopolymer- and hydrogel-based GPEs still exhibit a broad conductivity range, commonly spanning from 10^–5^ to 10^–2^ S cm^–1^, depending on factors such as composition, salt concentration, plasticizer or solvent retention, and gel architecture.^9,12^

Among these materials, sodium alginate stands out as a particularly attractive matrix for the development of biobased green GPE system because of its biodegradability, low cost, water solubility, and high density of polar functional groups, especially hydroxyl and carboxylate moieties, which can favor ion coordination.^13^ However, electrolytes based exclusively on alginate still show limitations in ionic transport, mainly associated with insufficient salt dissociation and restricted charge-carrier mobility within the polymer matrix, which ultimately results in relatively low ionic conductivity.^14^ This limitation hinders their direct application in modern devices such as supercapacitors and batteries, where fast ion transport and stable electrochemical performance are essential.^15^

Strategies capable of enhancing salt dissociation and ion–polymer interactions are therefore required to increase the density and mobility of charge carriers within the polymer matrix.^16^ In this context, the incorporation of lignin emerges as a promising approach. Lignin, an abundant aromatic biopolymer derived from the pulp and paper industry and bioethanol production, exhibits a high density of polar functional groups, such as phenolic and aliphatic hydroxyls, carboxyl groups, and methoxyl groups.^17^ These groups can act as interaction sites for ionic species, potentially favoring salt dissociation and contributing to an increased concentration of mobile ions.^18^ In addition, its predominantly amorphous nature can reduce the structural organization of the polymer matrix, increasing the amorphous fraction and free volume, which are key factors facilitating charge carrier migration.

Despite these characteristics, lignin in its native form exhibits predominantly insulating behavior and does not directly contribute to electrical conduction, although it can modify the chemical and structural environment of the system.^16^ In this sense, the introduction of ionic species into lignin-containing polymer systems represents an effective strategy to enhance ionic transport in renewable polymer electrolytes.^9^

In this context, the incorporation of sodium sulfate (Na_2_SO_4_) into the polymer matrix enables the establishment of ionic conduction by introducing mobile ionic species and defining the ionic content of the system. The ionic conductivity is therefore governed by the balance between salt concentration and ion mobility within the polymer network. At moderate concentrations, the presence of Na_2_SO_4_ increases the number of charge carriers available for transport, while excessive salt content may promote ionic association, reducing the effective fraction of mobile ions. In parallel, the polar functional groups present in alginate and lignin provide a suitable environment for ion accommodation within the polymer matrix, enabling the formation of continuous pathways for ionic transport, consistent with the behavior expected for biobased green GPEs.

To the best of our knowledge, the combined use of sodium alginate, lignin, and sodium sulfate (Na_2_SO_4_) in gel polymer electrolyte systems remains unexplored. While alginate-based electrolytes and lignocellulosic materials have been individually investigated,^6,19^ the integration of lignin as a functional component in alginate-based electrolytes, together with sodium sulfate as ionic source, has not been systematically reported.

From this perspective, the present work proposes a biobased green GPE system in which ionic transport is governed by the interplay between salt concentration, polymer–ion interactions, matrix organization, and viscoelastic properties. To validate this approach, a systematic investigation was performed using complementary spectroscopic, rheological, electrochemical, and dielectric analyses, including Fourier transform infrared spectroscopy (FTIR), oscillatory rheology, electrochemical impedance spectroscopy (EIS), transference number measurements, linear sweep voltammetry (LSV), cyclic voltammetry (CV), and dielectric response evaluation.

Particular attention is given to understanding the relationship between ionic dissociation, charge carrier density, and transport mechanisms within the polymer matrix. The performance of the developed electrolytes is assessed in terms of ionic conductivity, ion transference number, electrochemical stability window, and charge transport behavior. Furthermore, the practical applicability of the proposed system is qualitatively demonstrated through a simple electrochemical device, highlighting its potential for sustainable energy storage applications such as supercapacitors and batteries.

## Materials and Methods

### Materials

Sodium Alginate P.A. ((C_6_H_7_NaO_6_)_n_, 90% purity, Dinâmica Química Contemporânea Ltd.a., Indaiatuba, SP, Brazil) was used as the polymer matrix for the preparation of gel polymer electrolytes. Anhydrous Sodium Sulfate P.A. (Na_2_SO_4_, 99% purity, MW 142.04, LabSynth, Diadema, SP, Brazil) was employed as the ionic salt to provide mobile charge carriers. Kraft lignin powder derived from *Eucalyptus* spp. Was used as a functional additive and was supplied by Suzano S.A. (SP, Brazil). Sodium Hydroxide Micropearls P.A. (NaOH, 98% purity, MW 40.00, LabSynth, Diadema, SP, Brazil) was used for pH adjustment during lignin treatment. All reagents were used as received without further purification, and distilled water was used as the solvent in all experiments.

### Preparation of Gel Polymer Electrolytes

Gel polymer electrolytes were prepared to evaluate the influence of lignin incorporation and salt concentration on ionic transport properties.

Initially, 1 g of sodium alginate was dissolved in 25 mL of distilled water under continuous magnetic stirring until a homogeneous solution was obtained. A salt-containing formulation was prepared by adding 0.136 g of Na_2_SO_4_ to the alginate solution under stirring until complete dissolution.

For the lignin-containing formulations, Kraft lignin was first dispersed in 25 mL of distilled water containing Na_2_SO_4_. The mixture was maintained under magnetic stirring in a water bath at 70 °C for 3 h to promote dissolution and improve dispersion. After this period, the pH was adjusted to approximately 10 using NaOH solution to enhance lignin compatibility with the polymer matrix.

The lignin content was defined from a preliminary screening step. Different Kraft lignin masses (0.177, 0.277, and 0.377 g) were evaluated under the same preparation conditions, while the Na_2_SO_4_ amount was kept constant at 0.136 g as a reference condition. Among the tested formulations, the system containing 0.277 g of lignin, corresponding to 27.7 wt % relative to sodium alginate, exhibited the highest ionic conductivity.

Subsequently, the lignin-containing solution was incorporated into the alginate matrix to form the gel polymer electrolytes. Based on this result, the lignin content was fixed at 27.7 wt %, while the Na_2_SO_4_ content was varied to evaluate the effect of salt concentration on ionic transport independently from changes in lignin content.

All samples were prepared under identical experimental conditions. The compositions of the prepared gel polymer electrolytes and their corresponding sample codes are summarized in Table 1. For clarity, additive contents are expressed as wt % relative to the sodium alginate mass.

**Table 1:** Formulations of Sodium Alginate-Based Gel Polymer Electrolytes Containing Lignin and Na_2_SO_4_ ^a^

sample	lignin (wt %)	Na_2_SO_4_ (wt %)
Alg	0.0	0.0
Alg-Na	0.0	13.6
Alg-Lig-Na20.6	27.7	20.6
Alg-Lig-Na26.6	27.7	26.6
Alg-Lig-Na33.6	27.7	33.6

a Additive contents are also expressed as wt.% relative to the sodium alginate mass.

### Characterization

#### Fourier Transform Infrared Spectroscopy (FTIR)

FTIR spectra were recorded using an IRPrestige-21 spectrometer (Shimadzu, Kyoto, Japan) in the wavenumber range of 500–4000 cm^–1^ with a resolution of 1 cm^–1^. The spectra were used to identify functional groups and evaluate intermolecular interactions within the polymer electrolyte system.

#### UV–vis Spectroscopy

Optical properties were evaluated using a UV–vis spectrophotometer (UV-M51, Bel Engineering, Monza, Italy) in the wavelength range of 300–800 nm. Transmittance measurements were used to assess structural homogeneity and optical behavior of the gels.

#### Rheological Characterization

The rheological behavior of the gel polymer electrolytes was investigated using a stress-controlled rheometer (AR-G2, TA Instruments, New Castle, DE, USA) equipped with parallel plate geometry. Oscillatory measurements were performed using plates with a diameter of 25 mm under controlled conditions.

Frequency sweep tests were carried out over an angular frequency range of 0.1–100 rad s^–1^, at a constant strain within the linear viscoelastic region (LVR), previously determined by strain sweep experiments. All measurements were conducted at 25 °C to ensure thermal stability.

The storage modulus (*G*′) and loss modulus (*G*″) were obtained from small-amplitude oscillatory shear (SAOS) measurements. The complex viscosity (η*) was calculated according to

1 $$\eta^{*}=\frac{\sqrt{{\left(G^{\prime }\right)}^{2}+{\left(G^{\prime \prime }\right)}^{2}}}{\omega }$$

where *G*′ is the storage modulus, *G*″ is the loss modulus, and ω is the angular frequency. The loss tangent (tan δ), which represents the ratio between viscous and elastic contributions, was calculated as

2 $$\tan\delta =\frac{G^{\prime \prime }}{G^{\prime }}$$

where *G*′ is the storage modulus, *G*″ is the loss modulus, and ω is the angular frequency.

#### Electrochemical Characterization

Electrochemical analyses were performed using a potentiostat/galvanostat equipped with an impedance analyzer (IVIUM CompactStat, Ivium Technologies B.V., Eindhoven, The Netherlands). The electrolyte was assembled in a Teflon cell between two well-polished stainless-steel blocking electrodes (1.0 cm in diameter) separated by a fixed distance of 0.3 cm. All measurements were carried out in triplicate at room temperature.

Electrochemical impedance spectroscopy (EIS) was carried out in the frequency range from 10^6^–10^–1^ Hz with a 5 mV perturbation amplitude. The bulk resistance (*R*_*b*_) was obtained from the intercept of the Nyquist plot with the real axis in the high-frequency region, and the ionic conductivity (σ) was calculated according to eq 3

3 $$\sigma =\frac{L}{R_{b}S}$$

where *L* is the electrolyte thickness, *S* is the electrode–electrolyte contact area, and *R*_*b*_ is the bulk resistance.

The dielectric constant (ε′) and dielectric loss (ε″) were calculated from the complex impedance data using eqs 4 and 5

4 $$\varepsilon^{\prime }=\frac{Z^{\prime \prime }}{\omega C_{0}\left(Z^{\prime 2}+Z^{\prime \prime 2}\right)}$$

5 $$\varepsilon^{\prime \prime }=\frac{Z^{\prime }}{\omega C_{0}\left(Z^{\prime 2}+Z^{\prime \prime 2}\right)}$$

where *Z*′ and *Z*″ are the real and imaginary parts of the impedance, respectively, ω is the angular frequency (ω = 2π*f*), and *C*_0_ is the vacuum capacitance.

To analyze dielectric relaxation and minimize electrode polarization effects, the real (*M*′) and imaginary (*M*″) components of the electric modulus were calculated according to eqs 6 and 7

6 $$M^{\prime }=\frac{\varepsilon^{\prime }}{\varepsilon^{\prime 2}+\varepsilon^{\prime \prime 2}}$$

7 $$M^{\prime \prime }=\frac{\varepsilon^{\prime \prime }}{\varepsilon^{\prime 2}+\varepsilon^{\prime \prime 2}}$$

The dielectric loss tangent was calculated using eq 8

8 $$\tan\delta =\frac{\varepsilon^{\prime \prime }}{\varepsilon^{\prime }}=\frac{M^{\prime \prime }}{M^{\prime }}$$

The ionic (*t*_ion_) and electronic (*t*_ele_) transference numbers were determined by DC polarization under an applied voltage of 1 V for 350 s. The transference numbers were calculated from the initial (*I*_*i*_) and steady-state (*I*_*f*_) currents using eqs 9 and 10

9 $$t_{ion}=\frac{I_{i}-I_{f}}{I_{i}}$$

10 $$t_{ele}=\frac{I_{f}}{I_{i}}$$

The ionic and electronic conductivities were then obtained from eqs 11 and 12

11 $$\sigma_{ionic}=t_{ion}\cdot \sigma$$

12 $$\sigma_{electronic}=t_{ele}\cdot \sigma$$

The electrochemical stability window was evaluated by linear sweep voltammetry (LSV) in the potential range of 0–4 V at a scan rate of 10 mV s^–1^. Cyclic voltammetry (CV) was performed between −1 and 1 V at a scan rate of 100 mV s^–1^ for 10 cycles to assess redox behavior and electrochemical stability.

## Results and Discussion

### FTIR Analysis

The FTIR spectra of sodium alginate, lignin, Na_2_SO_4_, and their corresponding GPEs are shown in Figure 1. The spectra exhibit the characteristic absorption bands of each component, confirming the successful incorporation of lignin and salt into the polymer matrix.

**Figure 1:**
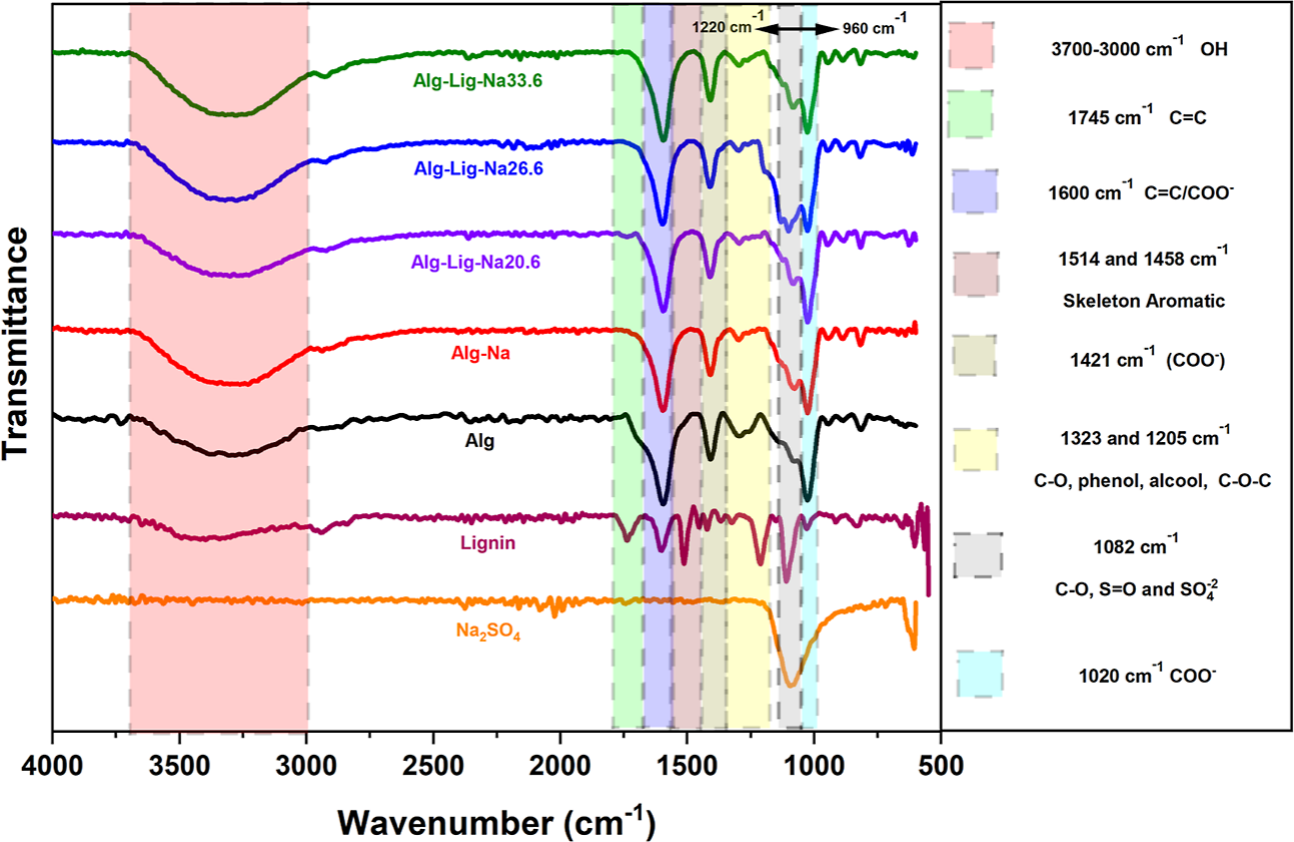
FTIR spectra of alginate-based gel polymer electrolytes with varying lignin and Na_2_SO_4_ contents. The highlighted regions correspond to O–H, carbonyl/aromatic, carboxylate, and fingerprint bands. Changes in band profiles indicate interactions between the polymer matrix, lignin, and ionic species.

The broad band observed in the 3700–3000 cm^–1^ region is attributed to O–H stretching vibrations and is characteristic of alginate, lignin, and the gel polymer electrolytes, reflecting the presence of hydroxyl-containing groups and bound water in the polymeric matrix. Lignin also exhibits a distinct band at 1745 cm^–1^, assigned to C=O stretching vibrations of carbonyl groups, which is characteristic of this component.

Around 1600 cm^–1^, the asymmetric stretching of carboxylate groups (–COO^–^) from alginate overlaps with aromatic C=C vibrations from lignin. In the composite electrolytes, this region presents a combined spectral profile, indicating the coexistence of contributions from both components within the polymer matrix. Similarly, the bands at 1514 and 1458 cm^–1^, associated with aromatic skeletal vibrations of lignin, and the band at 1421 cm^–1^, attributed to the symmetric stretching of COO^–^ groups, remain within an overlapped region in the mixed systems, without the appearance of new distinct bands.

In the 1323–1205 cm^–1^ interval, bands related to C–O stretching vibrations from phenolic, alcoholic, and ether groups (C–O–C) are observed. In the composite formulations, this region becomes broader, indicating greater overlap of vibrational contributions from alginate and lignin functional groups. A similar effect is observed in the 1220–962 cm^–1^ region, particularly for Alg-Lig-Na26.6, where the band profile becomes wider and less defined. This behavior is consistent with the superposition of contributions from C–O and C–O–C groups of the polymeric components together with sulfate-related vibrations, suggesting modifications in the local ionic environment of the matrix.

The band near 1082 cm^–1^ is associated with sulfate-related vibrations from Na_2_SO_4_, although this region must be interpreted with caution due to overlap with polymer bands. Finally, the region between 945 and 815 cm^–1^ is attributed to glycosidic structures of sodium alginate, confirming the preservation of the main polysaccharide backbone in all electrolyte formulations.^20–22^

### UV–vis Transmittance Spectroscopy

The UV–vis transmittance spectra of the samples are presented in Figure 2. Overall, a decrease in transmittance is observed upon lignin incorporation and with increasing Na_2_SO_4_ content in the lignin-containing systems.

**Figure 2:**
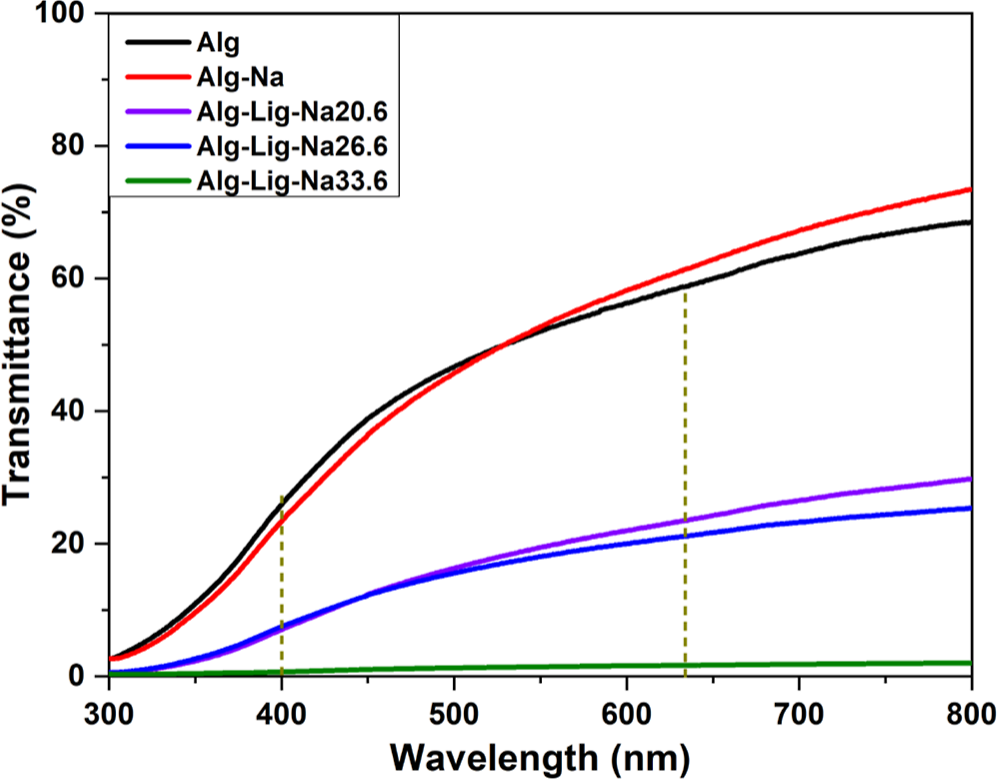
UV–vis transmittance spectra of alginate-based gel polymer electrolytes with different lignin and Na_2_SO_4_ contents. The dashed vertical lines indicate the wavelengths selected for comparison (400 and 633 nm).

The Alg and Alg-Na samples exhibit the highest transmittance values across the visible range, indicating greater optical transparency. This behavior can be attributed to the relatively homogeneous nature of the alginate matrix and the absence of highly absorbing aromatic structures, which facilitates light transmission through the material.^23^

At 633 nm, the Alg-Na sample shows slightly higher transmittance than pure alginate, suggesting that the addition of Na_2_SO_4_ does not disrupt the optical homogeneity of the system and may promote subtle rearrangements within the polymer network, likely associated with ionic interactions between Na^+^ and the alginate chains.

In contrast, the incorporation of lignin leads to a significant reduction in transmittance across the entire spectral range. This effect is clearly observed for Alg-Lig-Na20.6, Alg-Lig-Na26.6, and Alg-Lig-Na33.6, and becomes progressively more pronounced with increasing salt content. The decrease in transparency is consistent with the intrinsic light-absorbing nature of lignin, which contains aromatic and phenolic groups capable of absorbing radiation in both the ultraviolet and visible regions.^24^

At 400 nm, transmittance decreases from Alg-Lig-Na20.6 to Alg-Lig-Na33.6, indicating a progressive increase in light attenuation. A similar trend is observed at 633 nm, where the lignin-containing samples show a continuous reduction in transmittance with increasing salt content. Notably, Alg-Lig-Na33.6 exhibits near-zero transmittance throughout the visible range, indicating a highly opaque system.

In addition to the chromophoric nature of lignin, the reduction in transmittance with increasing salt concentration suggests modifications in the polymer matrix. FTIR spectra indicate overlapping contributions in the 1323–962 cm^–1^ region, associated with C–O, C–O–C, and sulfate-related vibrations. Although no new bands are observed, subtle variations in band profiles suggest interactions between alginate, lignin, and ionic species, which may contribute to light attenuation.^25^

Furthermore, the distinct separation between the curves of Alg-Lig-Na20.6 and Alg-Lig-Na26.6 suggests that relatively small changes in salt concentration can significantly affect the optical response of the system. This behavior indicates that the optical properties are sensitive to compositional variations and may serve as an indirect indicator of structural organization within the polymer matrix.

### Viscoelastic Behavior of GPEs

The viscoelastic properties of the GPEs were evaluated through oscillatory rheology, including storage modulus (*G*′), loss modulus (*G*″), complex viscosity (η*), and loss tangent (tan δ) as a function of angular frequency (ω), as shown in Figure 3.

**Figure 3:**
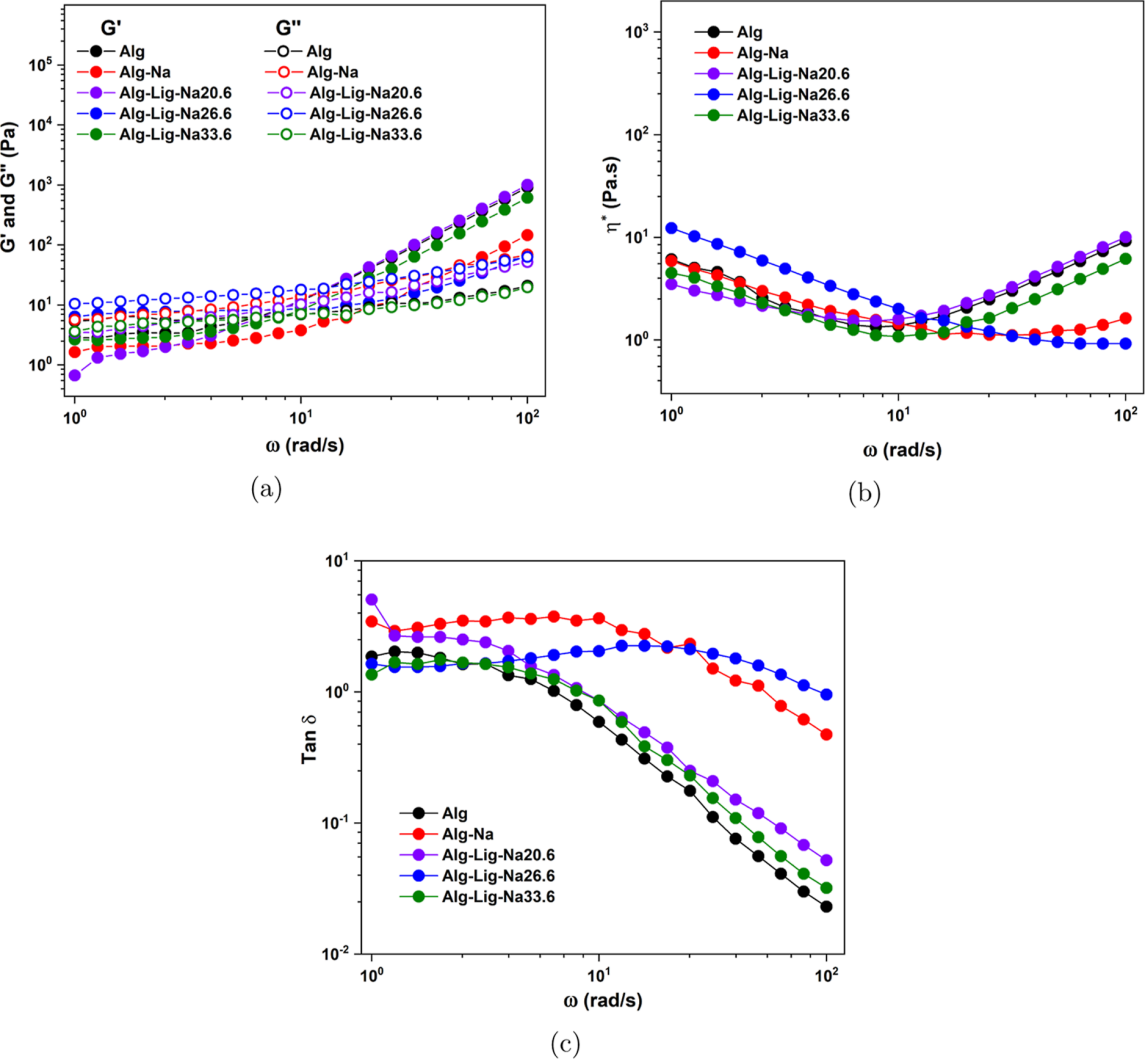
Rheological behavior of alginate-based gel polymer electrolytes with different lignin and Na_2_SO_4_ contents: (a) *G*′ and *G*″, (b) η*, and (c) tan δ as a function of angular frequency.

All systems exhibit frequency-dependent behavior, characteristic of physically cross-linked gels governed by reversible interactions, as shown in Figure 3. At low frequencies, *G*″ tends to be comparable to or higher than *G*′, indicating dominant viscous behavior (Figure 3a). As the frequency increases, *G*′ becomes more prominent, reflecting the transition to a more elastic response due to reduced time for structural relaxation.

The pure alginate system (Alg) shows relatively low *G*′ values and strong frequency dependence (Figure 3a), indicating a weak and highly dynamic network with limited structural stability. The addition of Na_2_SO_4_ (Alg-Na) increases both *G*′ and *G*″; however, *G*″ remains dominant over a wide frequency range, indicating that viscous dissipation still governs the mechanical response.

The incorporation of lignin significantly enhances the viscoelastic properties of the system. In particular, the Alg-Lig-Na20.6 composition exhibits the highest *G*′ values at intermediate and high frequencies (Figure 3a), indicating the formation of a more interconnected and mechanically robust network. This suggests that lignin acts as a multifunctional component, promoting additional physical interactions and reinforcing the polymer structure.

The complex viscosity (η*) curves further support this behavior (Figure 3b). The Alg-Lig-Na20.6 sample shows an increase in η* at higher frequencies, indicating resistance to deformation and structural integrity under dynamic conditions. In contrast, the Alg-Lig-Na26.6 system exhibits a continuous decrease in η*, suggesting weakening of the network due to excessive ionic interactions and possible structural disruption.

The loss tangent (tan δ) provides additional insight into the balance between elastic and viscous contributions (Figure 3c). The Alg-Lig-Na20.6 composition exhibits tan δ < 1 over a significant frequency range, indicating predominantly elastic behavior. This is a key characteristic for GPEs, as it reflects the ability of the material to maintain structural integrity while accommodating ionic transport. In contrast, the Alg-Na system maintains tan δ > 1 over most of the frequency range, indicating dominant viscous behavior and reduced mechanical stability.

At higher salt concentrations (Alg-Lig-Na26.6 and Alg-Lig-Na33.6), an increase in tan δ and a reduction in *G*′ and η* are observed (Figure 3a,b, and c), indicating weakening of the network due to electrostatic screening and saturation of coordination sites. This leads to increased molecular mobility but reduced structural cohesion.

Overall, the rheological analysis demonstrates that the Alg-Lig-Na20.6 composition provides the most favorable balance between elasticity and mobility, as evidenced by the combined trends in *G*′, η*, and tan δ shown in Figure 3a, b, and c. This balance is particularly important for electrochemical applications, as it ensures sufficient mechanical stability of the electrolyte while maintaining the segmental mobility required for ion transport, consistent with the strong relationship between rheological behavior and functional performance reported for polymeric systems.^26^ In this context, the rheological results suggest that the Alg-Lig-Na20.6 formulation may also exhibit a promising ionic conduction behavior, which is examined in the following section through ionic conductivity measurements.

### Ionic Conductivity

The ionic conductivity, σ, of the alginate-based gel polymer electrolytes was evaluated as a function of composition to assess its impact on ionic transport. The results are presented in Figure 4a. Conductivity was calculated using eq 3 from the bulk resistance (*R*_*b*_) obtained from the Nyquist plots shown in Figure 4b, as described in the Electrochemical Characterization section.

**Figure 4:**
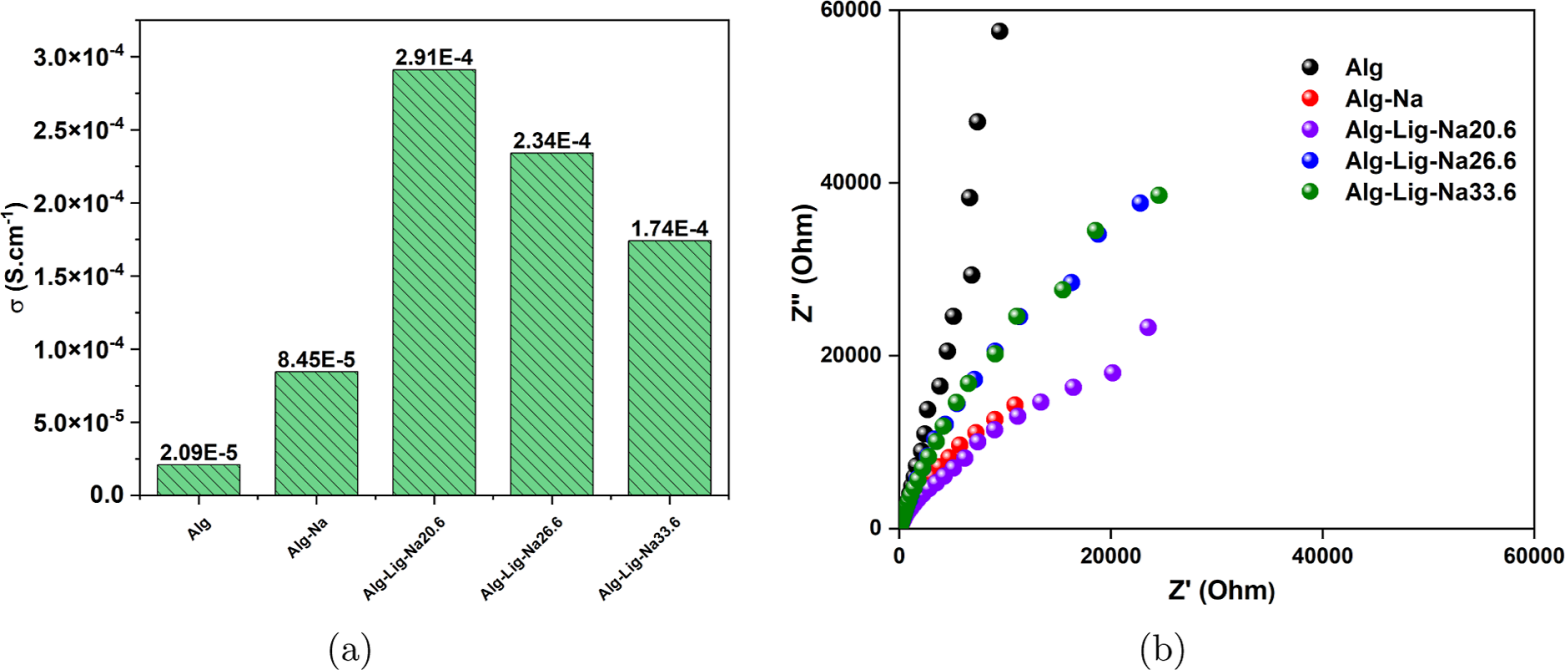
(a) Ionic conductivity (σ) of alginate-based GPEs with different lignin and Na_2_SO_4_ contents at room temperature. (b) Nyquist plots (Z″ vs Z′) of the corresponding samples obtained from EIS. The variation in conductivity is consistent with changes in ionic transport properties as a function of composition, with lignin-containing systems showing enhanced conductivity up to an optimal salt content.

In Figure 4a, the pristine alginate sample (Alg) exhibited an ionic conductivity of 2.09 × 10^–5^ S cm^–1^, which can be attributed to the limited mobility of ionic species associated with the carboxylate groups (–COO^–^) present in the polymer backbone. The addition of Na_2_SO_4_ (Alg-Na) increased the conductivity to 8.45 × 10^–5^ S cm^–1^, indicating the introduction of additional charge carriers into the system.

The incorporation of lignin led to a significant enhancement in ionic conductivity compared with the lignin-free formulations. The sample Alg-Lig-Na20.6 exhibited a conductivity of 2.91 × 10^–4^ S cm^–1^, which was the highest value among the investigated compositions, whereas Alg-Lig-Na26.6 showed a lower conductivity of 2.34 × 10^–4^ S cm^–1^. This behavior can be attributed to the presence of polar functional groups in lignin, which provide additional coordination sites for Na^+^ ions and modify the local ionic environment of the polymer matrix, thereby favoring ionic transport.

At higher salt concentration, as observed for Alg-Lig-Na33.6 (1.74 × 10^–4^ S cm^–1^), a reduction in conductivity occurs. This behavior suggests that, although the number of charge carriers increases, their effective mobility becomes limited due to stronger ionic interactions, such as ion pairing or aggregation, which restrict charge transport.

When compared with values reported in the literature (Table 2), the value obtained in this work is higher than those reported for sodium alginate systems containing LiBr or NH_4_Br, as well as for alginate/PVA-based electrolytes with K^+^ charge carriers, and is close to the highest conductivity reported for alginate/PVA systems containing Li^+^. These results indicate that the incorporation of lignin, combined with an appropriate Na_2_SO_4_ content, is an effective strategy for enhancing ionic transport in alginate-based GPEs.

**Table 2:** Comparison of Ionic Conductivity Values Reported for Alginate-Based GPEs, Including the Best-Performing Formulation from the Present Study

electrolytic system	σ (S cm^–1^)	reference
sodium alginate + LiBr	7.46 × 10^–5^	Shetty et al.^27^
sodium alginate/PVA + NH_4_Br	1.66 × 10^–5^	Jansi et al.^28^
alginate/PVA + K^+^	1.31 × 10^–5^	Wahab et al.^14^
alginate/PVA + Li^+^	3.31 × 10^–4^	Samsudin et al.^15^
alg-Lig-Na20.6 (this work)	2.91 × 10^–4^	This work

Regarding the Nyquist plots, Figure 4b, they exhibit a predominantly diffusive behavior, characterized by the absence of a well-defined semicircle in the high-frequency region and a linear trend at low frequencies. The absence of a semicircle suggests dominant bulk ionic conduction and negligible charge-transfer resistance at the electrode/electrolyte interface, whereas electrode polarization effects become more evident in the low-frequency dielectric response. This response is typical of gel polymer electrolytes, where ionic transport is governed by ion diffusion through the hydrated polymer network combined with segmental motion of the polymer chains.^29^

A progressive shift of the spectra toward lower Z′ values is observed with the incorporation of Na_2_SO_4_ and lignin, indicating a reduction in bulk resistance and an increase in ionic mobility. In the low-frequency region, the linear slope is associated with Warburg-type impedance, confirming that ion transport is controlled by diffusion processes within the polymer matrix.^30^

This trend is consistent with the UV–vis results, where a progressive decrease in transmittance was observed for lignin-containing samples with increasing salt content. The reduced transparency suggests increased light attenuation associated with changes in the internal organization of the system, which may also influence ion transport.

To further elucidate the mechanisms governing ionic transport, the dielectric properties are analyzed in the following section, providing insight into energy storage and dissipation under alternating electric fields and their relation to charge carrier dynamics.

### Dielectric Constant and Dielectric Loss

The dielectric properties of the polymer electrolytes were evaluated by analyzing the frequency dependence of the dielectric constant (ε′) and dielectric loss (ε″), as shown in Figure 5. These parameters were derived from the same impedance data used in the EIS analysis discussed in the previous section and were calculated according to eqs 4 and 5.

**Figure 5:**
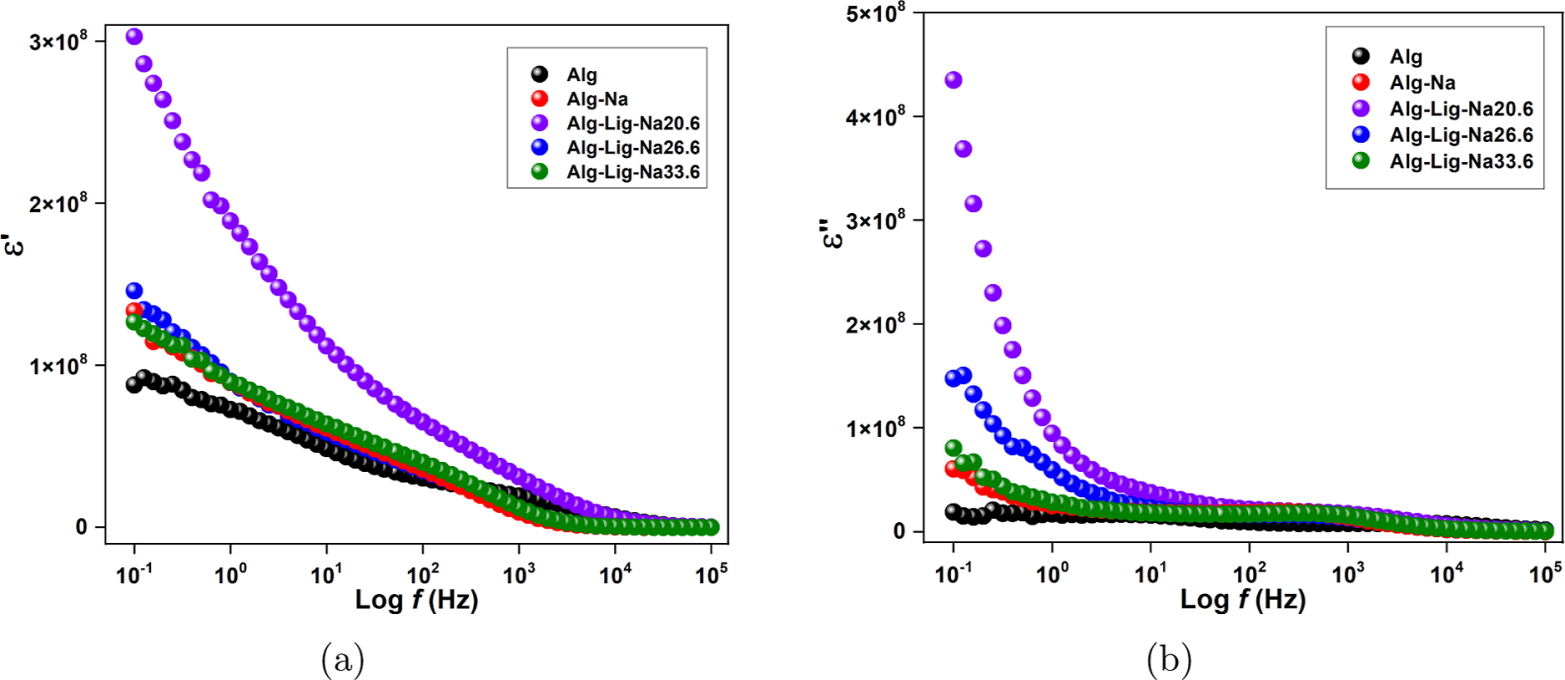
Frequency dependence of (a) ε′ and (b) ε″ for alginate-based GPEs with different lignin and Na_2_SO_4_ contents.

As shown in Figure 5a, all samples exhibit high ε′ values at low frequencies, followed by a continuous decrease as the frequency increases. This behavior is characteristic of polymer electrolytes and is mainly attributed to interfacial polarization (Maxwell–Wagner-Sillars effect), which becomes more pronounced at low frequencies due to the accumulation of charge carriers at the electrode/electrolyte interface.^31^

The pristine alginate sample (Alg) presents the lowest ε′ values over the entire frequency range, indicating a lower density of mobile charge carriers and limited polarization capability. The incorporation of Na_2_SO_4_ (Alg-Na) results in an increase in ε′, reflecting the introduction of additional ionic species into the system.

A more significant enhancement is observed for lignin-containing electrolytes. In particular, the sample Alg-Lig-Na20.6 exhibits the highest ε′ values at low frequencies, indicating a greater ability to store electrical energy. This behavior suggests an increased concentration of mobile ions and stronger polarization effects, which are consistent with the higher ionic conductivity observed for this composition.

The dielectric loss (ε″), shown in Figure 5b, follows a similar trend, with higher values at low frequencies and a gradual decrease as frequency increases. This behavior is associated with energy dissipation processes related to ionic conduction and charge carrier motion.^30^

Among the investigated samples, Alg-Lig-Na20.6 exhibits the highest ε″ values at low frequencies, indicating enhanced ionic mobility and increased energy dissipation due to more active charge transport processes. In contrast, the Alg sample shows the lowest ε″, confirming its reduced ionic mobility.

At higher frequencies, both ε′ and ε″ converge to lower values for all compositions, indicating that dipoles and ionic species are no longer able to follow the rapid oscillations of the applied electric field. This transition reflects the reduced contribution of polarization mechanisms and highlights the dominance of intrinsic material properties at high frequencies.

Although dielectric analysis and ionic conductivity provide important information on polarization behavior and charge transport, both approaches are strongly influenced by electrode polarization effects, particularly at low frequencies. As a result, intrinsic relaxation processes and bulk ionic dynamics cannot be clearly distinguished. To overcome this limitation, the electric modulus formalism is analyzed in the following section.

### Electrical Modulus

Figure 6 shows the frequency dependence of the real (*M*′) and imaginary (*M*″) components of the electric modulus for the different electrolyte compositions. The values of *M*′ and *M*″ were calculated according to eqs 6 and 7.

**Figure 6:**
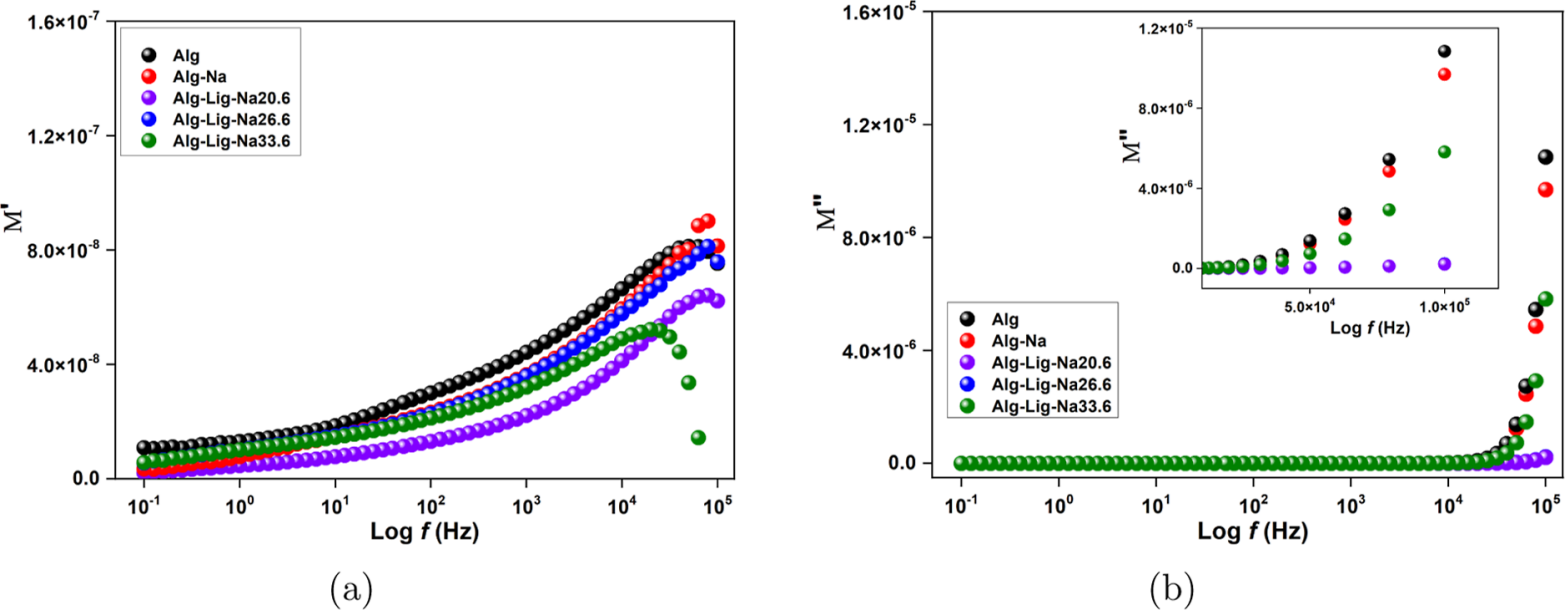
Frequency dependence of (a) real part (*M*′) and (b) imaginary part (*M*″) of the electric modulus for alginate-based GPEs with different lignin and Na_2_SO_4_ contents.

In general, *M*′ values increase with frequency (Figure 6a), reaching higher values in the high-frequency region. This behavior indicates that electrode polarization effects are progressively suppressed, allowing the intrinsic bulk relaxation processes of the material to dominate the electrical response.^32,33^

At low frequencies, *M*′ values approach zero for all samples, confirming that electrode polarization has a negligible contribution in this representation. This behavior is commonly associated with non-Debye relaxation processes in polymer electrolytes, reflecting the distribution of relaxation times within the system.^34^

As shown in Figure 6b, the imaginary component *M*″ exhibits a noticeable increase at high frequencies for all samples. However, the Alg-Lig-Na20.6 sample presents significantly lower *M*″ values compared to the other compositions, indicating a reduced accumulation of charge carriers and shorter relaxation times. This behavior suggests more efficient ionic dynamics and enhanced charge carrier mobility within the polymer matrix.

The combined behavior of lower *M*′ and suppressed *M*″ response for Alg-Lig-Na20.6 indicates more efficient ionic transport, where charge carriers can move with reduced hindrance and minimal energy loss associated with relaxation processes. This result is consistent with the improved ionic conductivity and transference number observed for this composition, confirming the beneficial role of lignin and salt in optimizing the polymer network.

Although the electric modulus, dielectric constant, and dielectric loss analyses provide important information about charge carrier relaxation, ionic transport within the polymer matrix, and energy storage behavior, a more complete interpretation of dielectric energy dissipation is still needed. This aspect is examined in the following section through the analysis of the loss tangent.

#### Loss Tangent Analysis

The dielectric loss tangent (tan δ) was analyzed as a function of frequency for the different GPEs, as shown in Figure 7. The loss tangent, defined as the ratio between dielectric loss (ε″) and dielectric constant (ε′), was calculated according to eq 8.

**Figure 7:**
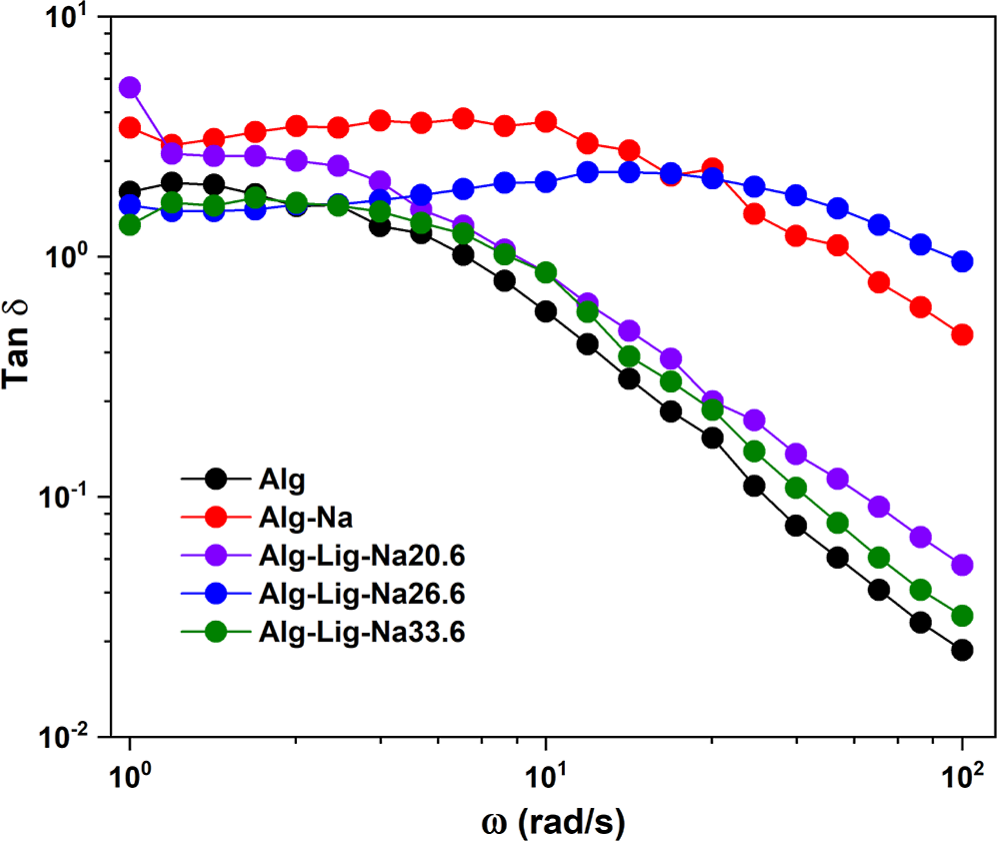
Frequency dependence of tan δ for alginate-based GPEs with different lignin and Na_2_SO_4_ contents. The pronounced increase at high frequencies reflects increased dielectric energy dissipation and highlights the influence of composition on charge carrier response.

At high frequencies (above ∼10^4^ rad/s), a pronounced increase in tan δ is observed for all samples. This behavior indicates that dipoles and charge carriers are no longer able to follow the rapid oscillations of the applied electric field, leading to increased energy dissipation. No well-defined relaxation peak is observed within the measured frequency range, suggesting a broad distribution of relaxation times in the system.

Comparing the different formulations, the pristine alginate sample (Alg) exhibits the lowest tan δ values across the entire frequency range, indicating limited charge carrier response. The addition of Na_2_SO_4_ (Alg-Na) results in a moderate increase in tan δ, reflecting the introduction of mobile ionic species.

A more significant increase in tan δ is observed for lignin-containing electrolytes. In particular, Alg-Lig-Na20.6 shows the highest values in the high-frequency region, indicating enhanced dielectric response associated with increased charge carrier mobility. For higher salt content (Alg-Lig-Na33.6), a slight reduction is observed, suggesting that excessive ionic concentration may limit effective mobility due to increased ionic interactions.

These results are consistent with the trends observed in the electric modulus analysis, where the shift of *M*″ toward higher frequencies indicated reduced relaxation times. Together, the modulus and tan δ analyses confirm that ionic transport in these systems is governed not only by the number of charge carriers but also by their dynamic response within the polymer matrix.

While the dielectric analyses provide insight into the dynamic response and energy dissipation behavior of the system, it is also essential to determine the nature of charge transport. In this context, in the next section, transference number measurements were performed to distinguish between ionic and electronic contributions to the total conductivity.

### Transference Number Measurement

The current–time responses obtained from the DC polarization measurements are presented in Figure 8. All samples exhibit a rapid initial decrease in current followed by a quasi-steady-state region. This behavior is associated with the polarization of mobile ions at the electrode/electrolyte interface and the subsequent dominance of electronic conduction in the steady-state regime.

**Figure 8:**
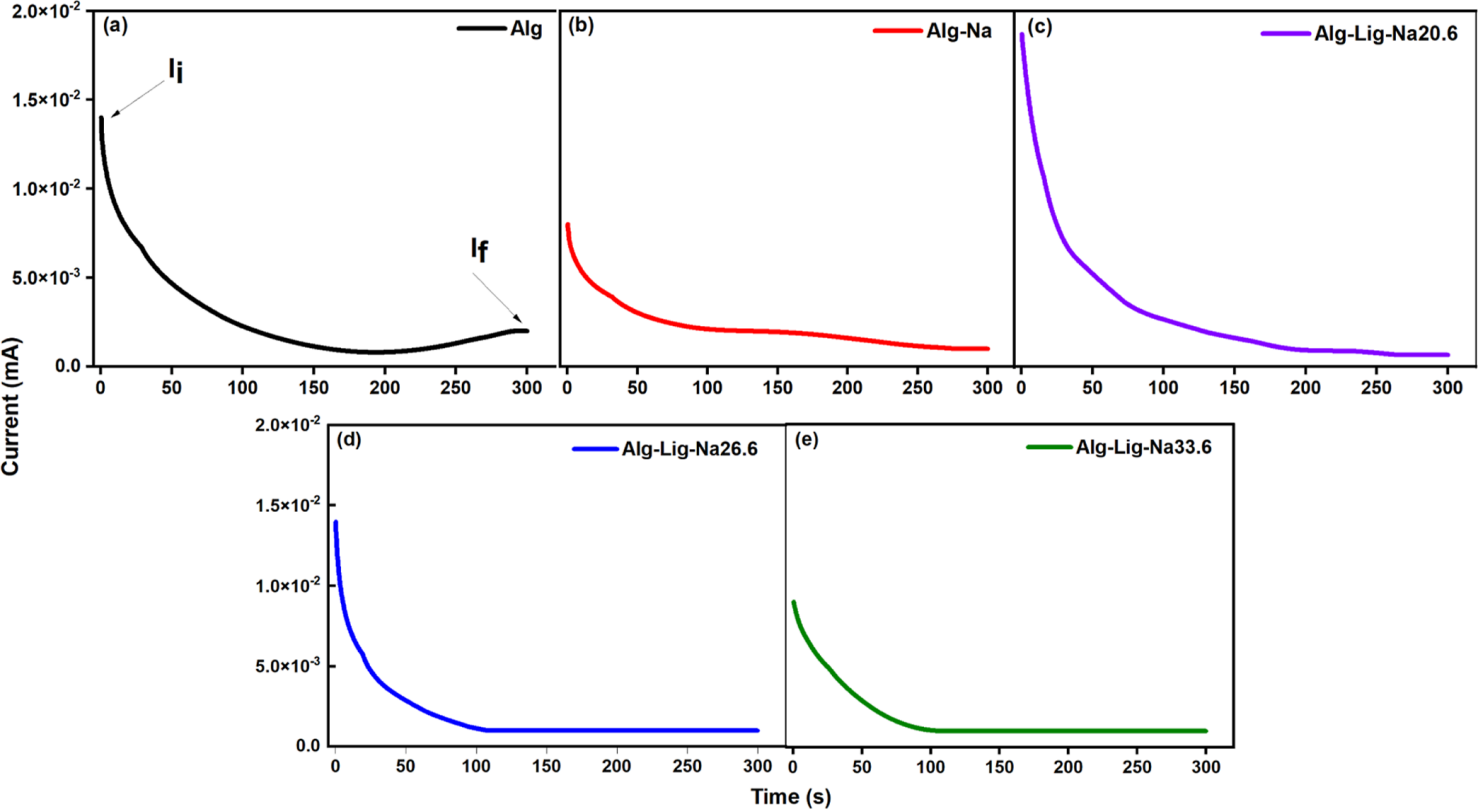
Current–time responses obtained from DC polarization measurements for alginate-based gel polymer electrolytes: (a) Alg, (b) Alg-Na, (c) Alg-Lig-Na20.6, (d) Alg-Lig-Na26.6, and (e) Alg-Lig-Na33.6. All samples exhibit an initial current decay followed by a quasi-steady-state regime, consistent with ionic polarization at blocking electrodes. The arrows indicate the initial (*I*_*i*_) and steady-state (*I*_*f*_) currents used for the calculation of transference numbers.

The ionic (*t*_ion_) and electronic (*t*_ele_) transference numbers were calculated using eqs 9 and 10, while the corresponding ionic and electronic conductivities were obtained using eqs 11 and 12. The results are summarized in Table 3.

**Table 3:** Transference Numbers and Partial Conductivities of the Alginate-Based GPEs, Including the Ionic Transference Number (*T*_ion_), Electronic Transference Number (*T*_ele_), Ionic Conductivity (σ_ionic_), and Electronic Conductivity (σ_electronic_)

sample	*t*_ion_	*t*_ele_	σ_ionic_ (S cm^–1^)	σ_electronic_ (S cm^–1^)
Alg	0.86	0.14	1.79 × 10^–5^	2.92 × 10^–6^
Alg-Na	0.87	0.12	7.35 × 10^–5^	1.01 × 10^–5^
Alg-Lig-Na20.6	0.98	0.05	2.85 × 10^–4^	1.45 × 10^–5^
Alg-Lig-Na26.6	0.92	0.07	2.15 × 10^–4^	1.50 × 10^–5^
Alg-Lig-Na33.6	0.89	0.11	1.54 × 10^–4^	1.91 × 10^–5^

All formulations exhibit predominantly ionic conduction, with *t*_ion_ values ranging from 0.86 to 0.98 and significantly lower *t*_ele_ values (0.05–0.14), confirming that ionic transport dominates the electrical response of the system. This behavior is desirable for electrochemical energy storage applications, where electronic leakage must be minimized.

This interpretation is consistent with recent reports on polysaccharide-based polymer electrolytes. For example, Cyriac et al.^35^ investigated biodegradable NaAlg/PVA electrolytes complexed with NaI and reported *t*_ion_ values higher than 0.9 for the optimized formulation, confirming that such systems tend to exhibit predominantly ionic conduction. The authors attributed this behavior to the strong coordination between alkali cations and the polar functional groups of the polymer matrix, which favors salt dissociation and enhances charge carrier mobility. A similar trend is observed in the present study, where the polar groups of alginate and lignin contribute to the predominance of ionic transport.

Among the studied compositions, the Alg-Lig-Na20.6 sample exhibits the best overall performance, presenting the highest ionic transference number (*t*_ion_ = 0.98) and the highest ionic conductivity (σ_ionic_ = 2.85 × 10^–4^ S cm^–1^). These results indicate that this formulation provides the most favorable balance between charge carrier concentration and mobility, leading to efficient ionic transport within the polymer matrix.

For higher salt content (Alg-Lig-Na33.6), a reduction in both *t*_ion_ and σ_ionic_ is observed, suggesting that excessive ionic concentration leads to increased ion–ion interactions and reduced mobility. This trend is consistent with the dielectric and conductivity analyses, reinforcing that optimal performance is achieved at intermediate salt content.

In the following section, the electrochemical stability of the developed electrolytes under applied potential is assessed by linear sweep voltammetry.

#### Linear Sweep Voltammetry (LSV) Analysis

The electrochemical stability of the GPEs was evaluated by LSV. The obtained voltammograms are presented in Figure 9.

**Figure 9:**
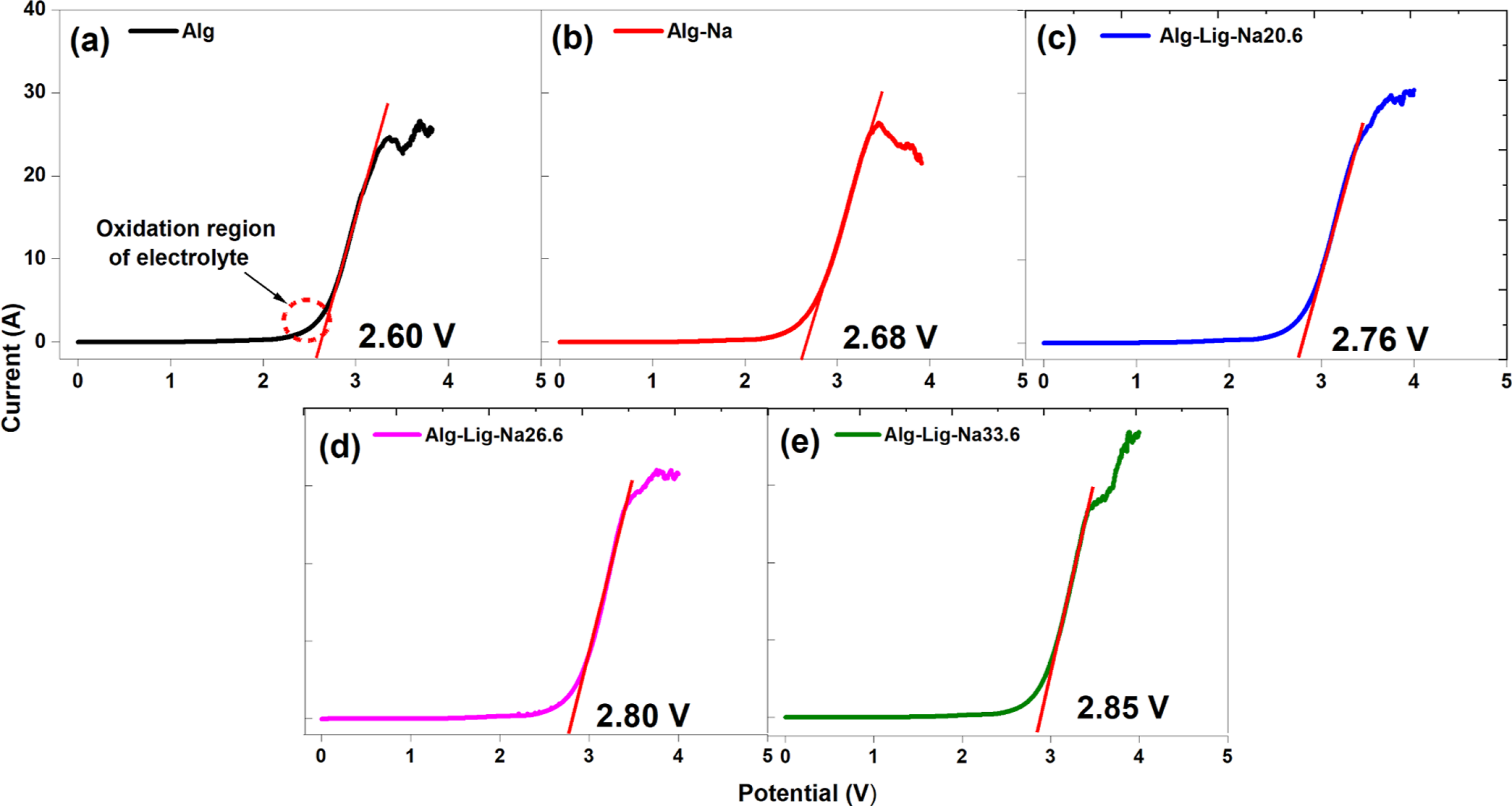
LSV curves of alginate-based GPEs: (a) Alg, (b) Alg-Na, (c) Alg-Lig-Na20.6, (d) Alg-Lig-Na26.6, and (e) Alg-Lig-Na33.6. The onset of the sharp current increase indicates the electrochemical decomposition potential of each formulation, determined from the intersection of the linear fit with the baseline region.

All samples exhibit a characteristic profile in which the current remains nearly constant at low potentials, followed by a sharp increase beyond a critical voltage. This behavior can be clearly observed in all panels (a–e) and is associated with the onset of electrolyte decomposition at the electrode/electrolyte interface, marking the limit of the electrochemical stability window.

The decomposition potential increases progressively with the modification of the polymer matrix. The pristine alginate electrolyte (Alg, Figure 9a) shows the lowest stability (2.60 V), indicating limited resistance to anodic polarization. The addition of Na_2_SO_4_ (Alg-Na, Figure 9b) slightly improves this value to 2.68 V. A more pronounced increase is observed for lignin-containing systems, reaching 2.76 V for Alg-Lig-Na20.6 (Figure 9c), 2.80 V for Alg-Lig-Na26.6 (Figure 9d), and a maximum of 2.85 V for Alg-Lig-Na33.6 (Figure 9e).

This systematic increase suggests that lignin incorporation enhances the structural stability of the polymer matrix under anodic conditions, likely by promoting stronger intermolecular interactions and reducing the susceptibility of the system to oxidative degradation.

An important observation is that the formulation exhibiting the highest ionic conductivity does not coincide with the one presenting the highest electrochemical stability. While Alg-Lig-Na20.6 (Figure 9c) showed the best conductive performance, Alg-Lig-Na33.6 (Figure 9e) exhibited the highest decomposition potential. This behavior highlights a trade-off between ionic transport and electrochemical stability.

This trade-off can be understood considering the interplay between ionic mobility and structural stabilization within the polymer matrix. At intermediate salt content (Alg-Lig-Na20.6), the system exhibits an optimal balance between charge carrier concentration and segmental mobility, resulting in enhanced ionic conductivity. This behavior is supported by rheological results, which indicate a more interconnected yet flexible network, and by dielectric analysis, which reveals higher dielectric loss and reduced relaxation times, both associated with improved ionic dynamics.

In contrast, at higher salt concentrations (Alg-Lig-Na33.6), stronger ion–ion and ion–polymer interactions promote increased structural rigidity of the polymer network, leading to improved electrochemical stability. However, these interactions also favor ion association and reduce segmental mobility, limiting effective ionic transport. Therefore, while intermediate salt content enhances ionic conductivity, higher salt concentrations improve resistance to electrochemical decomposition at the expense of ion mobility. This behavior is consistent with the typical conductivity-stability trade-off observed in polymer electrolytes.

#### Cyclic Voltammetry

The voltammograms obtained for the selected formulations are presented in Figure 10. All samples exhibit reproducible current–potential profiles over the consecutive cycles, indicating that the developed gel polymer electrolytes sustain stable electrochemical response within the investigated potential range.

**Figure 10:**
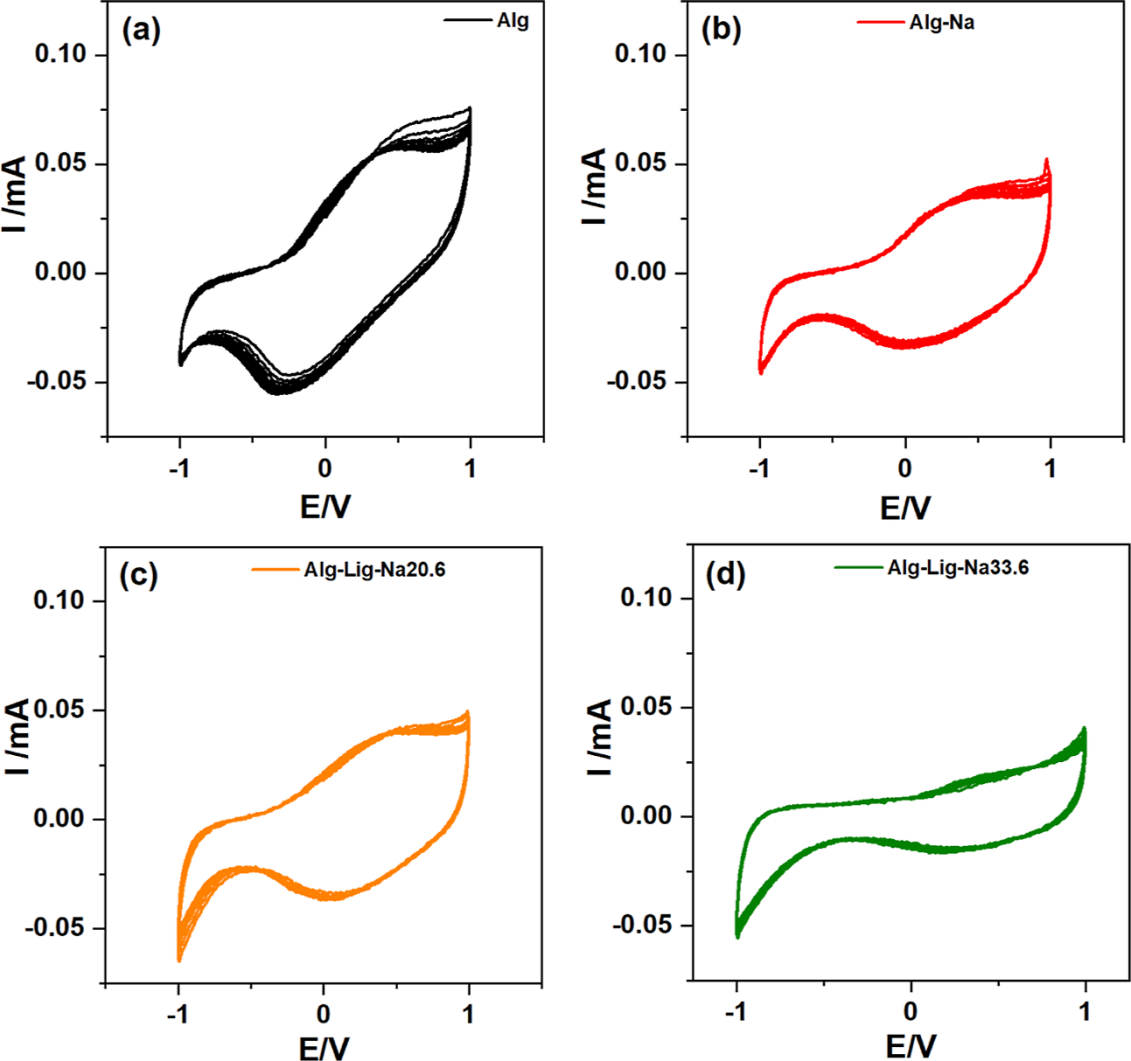
Cyclic voltammetry curves of alginate-based GPEs: (a) Alg, (b) Alg-Na, (c) Alg-Lig-Na20.6, and (d) Alg-Lig-Na33.6. The overlapping profiles over consecutive cycles indicate good electrochemical stability and reproducibility. Variations in current response and enclosed area reflect differences in ionic transport and charge storage behavior among the formulations.

All samples exhibit voltammetric profiles characterized by anodic and cathodic currents without sharp peaks, indicating predominantly capacitive behavior with contributions from reversible redox processes. This response confirms that the developed electrolytes are capable of sustaining ionic transport and enabling stable electrochemical activity.

Regarding cycling stability, only minor variations are observed across the 10 cycles for all formulations, indicating good electrochemical reversibility and structural stability of the polymer matrix. The pure alginate electrolyte (Figure 10a) shows a slight increase in voltammetric area, suggesting limited ionic transport capability due to the absence of additional ionic species.

The Alg-Na electrolyte (Figure 10b) exhibits a small reduction in voltammetric area over cycling, which may be associated with subtle rearrangements in the ionic environment and partial ion pairing effects.

The incorporation of lignin (Figure 10c) results in improved electrochemical response, with a slight increase in voltammetric area and more stable profiles over cycling. This behavior suggests enhanced ionic transport and improved interfacial charge transfer, consistent with the role of lignin in modifying the polymer network and promoting more favorable ion–polymer interactions.

In contrast, the formulation with higher salt content (Figure 10d) presents a reduced voltammetric area, indicating limitations in charge transport. This behavior is likely associated with increased ion–ion interactions and reduced ionic mobility at higher salt concentrations.

The stable voltammetric profiles may also suggest the formation of a relatively stable electrode/electrolyte interfacial environment during cycling. Considering the multicomponent nature of the electrolyte, composed of alginate, lignin, and Na_2_SO_4_, different organic functional groups and ionic species may contribute to interfacial stabilization under polarization. This behavior can be conceptually related to recently reported quasi-high-entropy solid electrolyte interphases, in which multiple components participate synergistically in the formation of stable and adaptive interfacial layers.^36^ However, the formation of a passivation layer cannot be directly confirmed from the present results and would require dedicated postcycling interfacial characterization, such as XPS analysis or surface-sensitive FTIR/ATR-FTIR measurements of the electrode after cycling.

It is worth noting that the formulation Alg-Lig-Na26.6 is not shown, as its voltammetric profile was very similar to that of Alg-Lig-Na20.6, without significant differences in shape or area. Therefore, for clarity, only representative compositions are presented.

Overall, the results indicate that the electrochemical performance of the GPEs depends on a balance between ionic concentration and polymer–ion interactions. Moderate salt content enhances ionic transport and electrochemical response, whereas excessive salt leads to reduced mobility due to ion aggregation effects.

The stability of the voltammograms over multiple cycles, combined with the absence of significant degradation, confirms that the developed electrolytes exhibit good electrochemical stability and reproducibility, supporting their potential application in energy storage devices.

#### Proof-of-Concept Device Demonstration

To provide an initial qualitative indication of the practical functionality of the developed gel polymer electrolytes, a simple proof-of-concept electrochemical device was assembled, as shown in Figure 11. In this configuration, the electrolyte was used to sustain electrical conduction under an external load (lamp), allowing a visual comparison of the charge transport capability of selected formulations.

**Figure 11:**
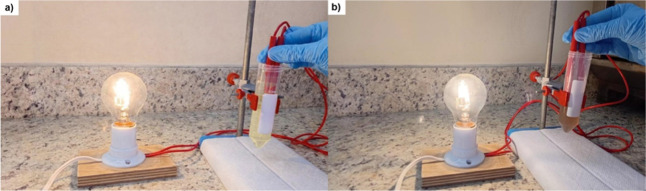
Proof-of-concept electrochemical device demonstrating the ability of the developed gel polymer electrolytes to sustain electrical conduction under an external load. (a) Alg-Lig-Na20.6 and (b) Alg-Na. The difference in light intensity qualitatively reflects variations in ionic conductivity among the formulations.

As observed in Figure 11a, the device employing the Alg-Lig-Na20.6 electrolyte exhibits visibly higher light emission, consistent with its higher ionic conductivity. In contrast, Figure 11b shows lower light intensity for the Alg-Na formulation, indicating more limited charge transport under the same demonstration conditions.

These observations qualitatively support the trends obtained from impedance, dielectric, and transference number analyses. Although this experiment does not represent a complete electrochemical cell performance evaluation, it provides a straightforward visual demonstration of the ability of the developed electrolytes to sustain electrical conduction in a simplified device configuration.

## Conclusion

In this work, biobased gel polymer electrolytes composed of sodium alginate, lignin, and Na_2_SO_4_ were successfully developed and systematically evaluated to elucidate the relationship between composition, ionic transport, and electrochemical performance.

The results demonstrate that ionic conductivity is governed by the balance between charge carrier concentration and mobility. The incorporation of Na_2_SO_4_ increases the number of mobile ions, while lignin modifies the polymer matrix by providing additional coordination sites and enhancing ion accommodation. As a result, a significant improvement in ionic conductivity was achieved, reaching 2.91 × 10^–4^ S cm^–1^ for the optimized composition (Alg-Lig-Na20.6), placing this system among the most competitive alginate-based electrolytes reported.

Dielectric and modulus analyses confirmed reduced relaxation times and improved charge carrier dynamics, while transference number measurements demonstrated predominantly ionic conduction (*t*_ion_ up to 0.98). Electrochemical stability increased with lignin and salt content, reaching 2.85 V, although a trade-off between conductivity and stability was observed.

From an application-oriented perspective, the obtained results provide clear guidelines for formulation selection. The intermediate salt composition (Alg-Lig-Na20.6), which exhibited the highest ionic conductivity, is more suitable for applications requiring efficient ionic transport, such as high-rate electrochemical devices. In contrast, formulations with higher salt content (e.g., Alg-Lig-Na33.6) are more appropriate for applications where enhanced electrochemical stability is required, even at the expense of reduced ionic mobility. Therefore, the selection of the optimal composition should be guided by the specific balance between conductivity and stability demanded by the target application.

Overall, the combination of sodium alginate, lignin, and Na_2_SO_4_ represents a simple, scalable, and effective strategy for designing sustainable GPEs with tunable properties. These findings provide valuable insights into ion transport mechanisms in biopolymer-based electrolytes and highlight their potential for application in energy storage devices.

## Data Availability

All data supporting the findings of this study are provided within the Article. Additional raw data (including original instrument outputs and analysis files) are available from the corresponding author upon reasonable request.

## Abbreviations

GPE: gel polymer electrolyte
SA: sodium alginate
Na_2_SO_4_: sodium sulfate
FTIR: fourier transform infrared spectroscopy
UV–vis: ultraviolet–visible spectroscopy
EIS: electrochemical impedance spectroscopy
LSV: linear sweep voltammetry
CV: cyclic voltammetry
TNM: transference number measurement
*R*_*b*_: bulk resistance
σ: ionic conductivity
ε′: dielectric constant
ε″: dielectric loss
*M*′: real part of electric modulus
*M*″: imaginary part of electric modulus
*t*_ion_: ionic transference number
*t*_ele_: electronic transference number.
